# Applications of MALDI-MS/MS-Based Proteomics in Biomedical Research

**DOI:** 10.3390/molecules27196196

**Published:** 2022-09-21

**Authors:** Laura Darie-Ion, Danielle Whitham, Madhuri Jayathirtha, Yashveen Rai, Anca-Narcisa Neagu, Costel C. Darie, Brînduşa Alina Petre

**Affiliations:** 1 Laboratory of Biochemistry, Department of Chemistry, “Alexandru Ioan Cuza” University of Iasi, Carol I bvd, No. 11, 700506 Iasi, Romania; 2 Biochemistry & Proteomics Laboratories, Department of Chemistry and Biomolecular Science, Clarkson University, 8 Clarkson Avenue, Potsdam, NY 13699, USA; 3 Laboratory of Animal Histology, Faculty of Biology, “Alexandru Ioan Cuza” University of Iasi, Carol I bvd, No. 22, 700505 Iasi, Romania; 4 Center for Fundamental Research and Experimental Development in Translation Medicine–TRANSCEND, Regional Institute of Oncology, 700483 Iasi, Romania

**Keywords:** proteomics, MALDI, tandem mass spectrometry (MS/MS), biomarkers, biomedical research

## Abstract

Matrix-assisted laser desorption/ionization (MALDI) mass spectrometry (MS) is one of the most widely used techniques in proteomics to achieve structural identification and characterization of proteins and peptides, including their variety of proteoforms due to post-translational modifications (PTMs) or protein–protein interactions (PPIs). MALDI-MS and MALDI tandem mass spectrometry (MS/MS) have been developed as analytical techniques to study small and large molecules, offering picomole to femtomole sensitivity and enabling the direct analysis of biological samples, such as biofluids, solid tissues, tissue/cell homogenates, and cell culture lysates, with a minimized procedure of sample preparation. In the last decades, structural identification of peptides and proteins achieved by MALDI-MS/MS helped researchers and clinicians to decipher molecular function, biological process, cellular component, and related pathways of the gene products as well as their involvement in pathogenesis of diseases. In this review, we highlight the applications of MALDI ionization source and tandem approaches for MS for analyzing biomedical relevant peptides and proteins. Furthermore, one of the most relevant applications of MALDI-MS/MS is to provide “molecular pictures”, which offer in situ information about molecular weight proteins without labeling of potential targets. Histology-directed MALDI-mass spectrometry imaging (MSI) uses MALDI-ToF/ToF or other MALDI tandem mass spectrometers for accurate sequence analysis of peptide biomarkers and biological active compounds directly in tissues, to assure complementary and essential spatial data compared with those obtained by LC-ESI-MS/MS technique.

## 1. Introduction

In 1976, Karas et al., discovered that the use of an energy-absorbent organic matrix could overcome the restrictive ionization and mass limitation of laser desorption MS technique that is used to analyze non-volatile polar biological and organic macromolecules [1,2]. This was a crucial step in the development of MALDI-based method by Koichi Tanaka and electrospray (ESI) ionization method by John Fenn; both scientists were awarded the 2002 Nobel Prize in Chemistry “*for their development of soft desorption ionisation methods for mass spectrometric analyses of biological macromolecules*” [3,4].

Since their discovery, both ESI and MALDI are the commonly used ionization methods for MS-based proteomic analysis, and the resulting data suggests a great deal of complementarity between ESI-MS and MALDI-MS [5]. MALDI is a “soft ionization” technique that produces rapid and effective ionization of a wide range of biomolecules (amino acids [6], peptides [5], proteins [7], oligonucleotides [8], oligosaccharides [9], and other organic molecules, such as small drugs [10]/metabolites [11] or large synthetic polymers [12]) using a matrix to absorb laser energy to produce ions with minimal fragmentation. The distinction between MALDI and ESI ionization sources is that the former produces low charged ions (singly and doubly protonated ions), which allows for easy molecular mass determination for most biomolecules [13] and easy interpretation of data comparative to ESI-MS [14].

The charged analytes/ions generated by MALDI source are then detected and measured using different types of mass analyzers, such as time-of-flight (ToF), quadrupole (Q), ion trap (IT), quadrupole ion trap (Q-IT), FT-ICR analyzers, etc. Coupling a quadrupole and ToF resulted in the production of high-resolution hybrid mass spectrometers, i.e., Q-TOF tandem mass spectrometric instrument [15]. For peptide and protein analysis, a MALDI source is commonly coupled with a ToF detector (MALDI-ToF) [16]. For example, for microbiological applications mainly, ToF analyzers are used [14]. Thus, during MALDI-ToF analysis, the ratio of mass-to-charge (*m*/*z*) of an ion is measured by determining the time required for it to travel the length of the flight tube [14]. For fast and quantitative analysis of small molecules, the MALDI source can be coupled with a triple quadrupole mass analyzer (MALDI-QQQ), resulting in a high correlation with data obtained by the traditional and well-established liquid chromatography (LC)-ESI-MS/MS analysis of routine biological assay samples [16]. A tandem mass spectrometry analysis of large peptides that can be used as biomarkers for various diseases involve MALDI coupled with a linear quadrupole ion trap mass analyzer (MALDI-Q-IT) [17].

A single-stage ESI-MS and MALDI-MS analysis are useful for determination of proteins and peptides molecular weight by the detection of *m*/*z* of related ions. Tandem MS is able to isolate a specific *m*/*z* (e.g., precursor ion) that can be subjected to dissociation (i.e., by collision-induced dissociation) followed by production of fragment or product ions, which offer information about molecular structure of the analyte (i.e., amino acid sequence of a peptide) [15]. A tandem mass spectrometer consists of two (MS/MS or MS^2^) mass analyzers connected in a series by collision cells, where ion fragmentation occurs, that take peptides analyzed in the first mass analyzer as intact molecules and breaks them in their constituents within the following mass analyzer [18]. Changes in the in-source voltages or the use of a linear ion trap instrument allows for more fragmentation than MS^2^ (MS^n^). MALDI-ToF MS is used for rapid determination of a mass pattern of proteins, while MALDI-ToF/ToF-MS/MS or liquid chromatography (LC) coupled with ESI-MS/MS is able to identify specific protein markers and peptide sequence variations among assessed species [19].

For a comprehensive analysis by ESI-MS, prior separation by LC is required, which is not necessary for MALDI-ToF MS analysis [14]. Thus, LC-MS/MS usually uses ESI as an ionization source (LC-ESI-MS/MS), this hyphenated technique combining the separation capability of high performance of liquid chromatography (HPLC) with tandem mass spectrometry (MS/MS) abilities in identification of small molecules with high-throughput, speed, and resolution [20,21]. However, many recent shotgun proteomics studies showed that LC-MALDI strategy allows high-quality data, which are often complementary to LC-ESI-MS/MS [22]. These LC-MS/MS approaches involving MALDI comprise the preparation of protein extracts, their enzymatic digestion, the separation of resulting peptides by nanoLC coupled to a collector, which deposits the micro-fraction onto a MALDI plate, followed by the MS analysis of the fractions [22]. MALDI can, however, limit the capacity to examine the largest macromolecular proteins, which in ESI form multiple charged ions and, only through a deconvolution process of these ions, the intact mass is attributed to the analyzed proteins [23,24].

Another approach, which combined the liquid chromatography tandem mass spectrometry (LCMS/MS) with high field asymmetric waveform ion mobility spectrometry (FAIMS), has been demonstrated to be effective for bottom-up proteomic investigations by increasing signal-to-noise and extending proteome coverage [25]. In comparison to earlier LESA FAIMS imaging workflows that used a planar FAIMS device, recent LESA FAIMS imaging workflows that integrated a cylindrical FAIMS device saw a considerable increase in the number of proteins discovered (over 10-fold for testes and kidney and 7-fold for brain) [26].

Caprioli et al., have shown that newly introduced MALDI timsToF imaging platform may provide the specificity required to separate components in intricate mixtures of lipid adducts while maintaining adequate data collecting rates (>2 pixels/s). High-performance TIMS separations combined with high-spatial-resolution and throughput imaging capabilities offer a uniquely adjustable platform to address numerous issues related to advanced molecular imaging applications [27].

A traditional proteomics approach based on MALDI-MS/MS or LC-ESI-MS/MS is usually applied for liquid biopsies, cell lysates, and tissue homogenates, allowing an accurate identification of complex protein extracts but do not provide fine correlation with in situ protein localization within tissue sections. Histology-directed MALDI-mass spectrometry imaging (MSI), also named “molecular microscopy”, provides “molecular pictures”, which offer quantitative and spatial information about proteins without labeling of potential targets as in immunohistochemistry (IHC) technique [28]. Due to a high molecular complexity, the direct structural analysis from tissue sections has shown limitation for intact protein identification [29]. Hence, a combinatorial proteomic workflow based on MALDI-MSI and on tissue extraction and analysis has been published to obtain successful identification and quantification of proteins while preserving their localization within the tissue sections [29]. Thus, to successfully map proteins directly on tissue sections, the MSI approach can be performed on MALDI-ToF/ToF or on a MALDI linear ion trap-Orbitrap (MALDI-LTQ-Orbitrap) instrument after absorbent matrix deposition [29]. Due to the limited number of proteins identified directly on tissue by MSI-related techniques, liquid micro-junction extraction experiments were performed followed by nanoLC-ESI-MS/MS analysis, as an efficient strategy to extract tryptic peptides and further identify the associated proteins of tissues [29]. Trypsin produces tryptic peptides by hydrolyzing proteins at the carboxyl side of the amino acids, lysine or arginine, making it the most popular serine protease employed in mass spectrometric bottom-up techniques. Studies have also documented the use of other enzymes, such as Arg-C, Asp-N, Chymotrypsin, Glu-C, and Lys-C, solo or in combination, to bottom-up proteins with less basic amino acid content [30].

Quantitative MALDI mass spectrometry imaging (Q-MSI) is a field with challenges due to a number of factors, such as (i) the co-crystallization of analyte-matrix crystals, variability in laser ablation (LA), and matrix ion suppression or (ii) high biological variability in samples combined with the limited sample cleanup and separation strategies available prior to MSI. However, many of these limitation were overcome by multiple reaction monitoring (MRM) suitable for pharmaceutical compounds with known molecular identity [31,32] or quantified by correlation with LC/MS/MS concentrations [33]. In Section 4, some of the main applications of MALDI-MS/MS and MSI performed on MALDI-MS/MS instruments will be discussed.

## 2. Ions Formation in MALDI Source

**Figure 1:**
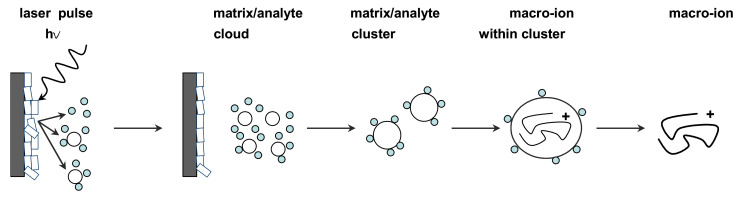
General representation of the desorption/ionization principle in MALDI-MS. Using a UV laser pulse, a matrix/analyte-particle cloud is desorbed from the co-crystalline matrix/sample solid solution deposited on a metal target. Proton-transfer from matrix ions is thought to be primarily responsible for the subsequent generation of analyte ions that are transferred to the mass analyzer and detected.

To generate protonated molecules in MALDI, a substantial excess of matrix solution is coprecipitated with the analyte by pipetting a submicroliter volume of the mixture onto a metal target plate and allowing it to dry at room temperature. Nanosecond laser pulses, usually from UV nitrogen lasers with a wavelength of 337 nm (and sometimes 335 nm UV lasers, as well as IR lasers) are then used to irradiate the resulting “solid solution”. Although the details of energy conversion and sample desorption/ionization are still being researched, the MALDI mechanism is represented and discussed in general below (Figure 1). 

The analyte ions formation is accomplished first by directing a pulsed laser beam onto the matrix/analyte homogenous “solid solution”. Some of the laser energy is absorbed by the matrix molecules causing effective vibrational excitation and disintegration of the “solid solution”, which further forms clusters of a single analyte molecule surrounded by neutral matrix molecules. Finally, the matrix molecules evaporate away from these clusters to leave the excited analyte molecules as an ionized species that is further electrostatically transferred into a mass analyzer. The ionization occurs in the first tens of nano seconds after irradiation and within the initial desorbing matrix/analyte cloud, and the analyte molecules may become ionized by simple protonation (M + H)^+^ or deprotonation (M-H)^−^ depending on the positive or negative ionization mode used for measurements. Charging analyte ions can be also generated by adduction of small ions, such as Na^+^, K^+^, acetate, and ammonium, present in MALDI solutions used for dissolving the organic matrix and the sample [34,35].

The MALDI matrix needs to fulfil some specific requirements, namely, (i) to be a non-volatile solid material that absorbs the laser radiation energy by possessing a conjugated aromatic ring structure, (ii) to minimize sample damage and facilitate the transfer of vibrational energy from the matrix to the sample in the vaporization process of the matrix, and (iii) to have a low sublimation rate to guarantee vacuum stability and be inert in terms of chemical reactivity to the analyte molecules. The molar ratio of matrix to analyte, the sample preparation technique, and the choice of the matrix determine the success of the MALDI measurements. The optimum matrix-to-analyte ratio is generally 10^2^–10^3^, strongly depending on the matrix used and the size of the analyte molecules [36,37].

The most employed sample preparation techniques on MALDI target are the dried droplet, fast evaporation, and multiple layering methods [38,39]. For reasons of solubility, proton transfer, and analyte ionization, there are specific organic matrix materials recommended for each biomolecule class analyzed by MALDI (Table 1). 

In addition to MALDI ionization source under vacuum conditions, atmospheric pressure (AP-MALDI) and liquid AP-MALDI were developed and applied to stabilize ion yields of multiple charged peptide and protein ions for a better sensitivity in MS [47,48]. In one minute of data acquisition per sample, Cramer et al. identified goat and sheep milk samples with 100% accuracy using liquid AP-MALDI [49].

Using MALDI ionization source for analyzing peptides and proteins, several advantages can be highlighted: (i) practical mass range of up to 300–500 kDa and low femtomole to low picomole sensitivity; (ii) tolerance of salt in millimolar concentration; and (iii) soft ionization with little to no fragmentation and suitability for analyzing a complex mixture. In contrast, MALDI may present also some disadvantages, such as (i) a matrix background is highly dependent on the matrix material employed and usually can be a problem for samples below a mass of 1000 Da; and (ii) a possibility of photo-chemical degradation of analyte molecules by UV/IR laser radiation [50].

## 3. Mass Analyzers for MALDI Ionization Source

Though the first mass analyzer, which used magnetic fields to separate ions, was found in the early 1900s, its application was limited to the chemical sciences. Modern mass analyzers, which are based on early magnetic technologies, provide great accuracy, sensitivity, mass range, and the capacity to provide structural information for large biological molecules, such as peptides and proteins. In addition, the sensitivity (pico to femtomole) required for many biomedical applications has been achieved with the MALDI ionization source, thanks in part to the high transmission efficiency of the mass analyzers [50].

Mass analyzers scan or select analyte ions over a particular range, measuring the ratio of mass-to-charge (*m*/*z*) of an ion, not its molecular mass. MALDI interfaces are used in a variety of ways with various mass analyzers. Time-of-flight (ToF) mass analyzers benefit from advancements in linear mode, reflectron, and orthogonal [51] ToF mass analyzers to achieve higher mass resolution and mass accuracy. Quadrupole ion traps, Fourier-transform ion cyclotron resonance (FTICR) instruments, and, more recently, Orbitrap [52] mass analyzers have supplemented and/or met the requirement for high-resolution accurate and high sensitivity mass spectrometry for analyzing the complexity of biological samples, such as proteome and metabolome [53,54]. Modern MS instruments are almost entirely hybrid, containing two or more different mass analyzers (represented by ToF/ToF, quadrupole-ToF and quadrupole-orbitraps, allowing rapid, accurate, and precise mass determination of precursor and product ions, a long-desired goal in MS.

ToF analyzers use a short voltage gradient to accelerate the ions and measure the time it takes them to pass over a field-free flight tube; the flight time being proportional to the square root of the m/z. Ion manipulations, such as delayed extraction of ions from the source, two stage sources with complex voltage gradients, and reflectron technology, are used to increase resolution, and a commercial ToF instrument can typically achieve resolution of 10,000 or greater [55]. 

**Figure 2:**
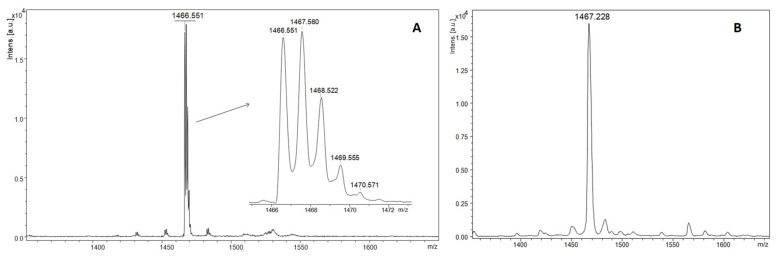
MALDI ToF mass spectra in reflectron (**A**) and linear (**B**) of intact ECP peptide with DHB as matrix.

Typical mass spectra for MALDI ToF analysis in linear and reflector mode are shown below for two variants of non-nitrated and nitrated ECP peptides fragment (H_2_N-AMRAINNYRWR-COOH). The MALDI-ToF mass spectrum of non-nitrated ECP peptide showed a single intact molecular [M + H]^+^ ion at 1466.551 with monoisotopic distribution in reflectron mode (Figure 2A) and average [M + H]^+^ ion at 1467.228, in low resolution linear mode (Figure 2B). 

**Figure 3:**
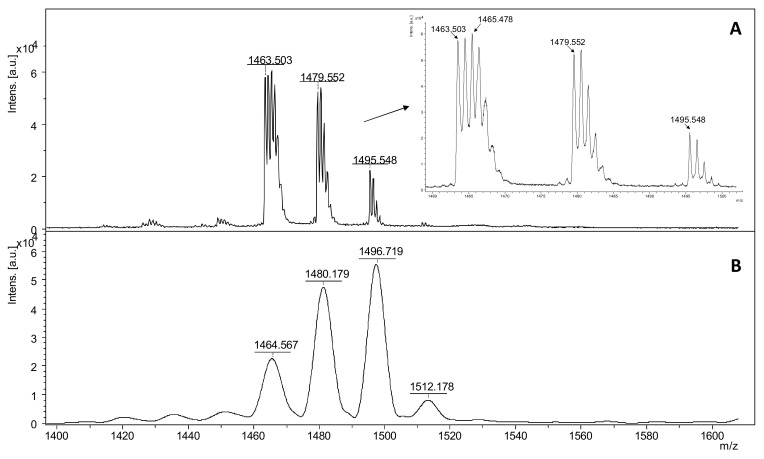
MALDI ToF mass spectra in reflectron (**A**) and linear (**B**) of intact ECP nitrated peptide with DHB as matrix where the NO_2_ group of nitro tyrosine is photcehmical degraded due to UV laser radiation in MALDI ionization source.

A characteristic addition of +45 Da to the molecular ion of the nitro-substituted tyrosine group in ECP at *m*/*z* 1495.548 can be observed in reflectron mode (Figure 3A), while in linear mode the same peptide fragment was assigned at 1496.719 as a single *m*/*z* signal (Figure 3B). The nitro tyrosine group undergoes specific photochemical fragmentation during UV laser radiation in MALDI-ToF MS, resulting in three major fragments [M + H]^+^-16 Da, [M + H]^+^-30 Da, and [M + H]^+^-32 Da. This specific fragmentation of 3-nitro-tyrosyl residues in peptides, clearly revealed by MALDI ToF reflectron mode, corresponds to the loss of one oxygen to form a 3-nitroso-Tyr derivative [Tyr(NO)], the loss of two oxygens to form a nitrene-type fragment [Tyr (:N:)], and the reduction in the nitro group to form an amine [Tyr (NH_2_)] [56].

In quadrupole ion traps, ions are focused into a small volume with an oscillating electric field; then, ions are resonantly activated and ejected by electronic manipulation of this field [57]. Affordable quadrupole ion traps depend primarily on radio frequency (RF) fields and have proven very useful in mass spectrometric data in high complexity sample analysis because they can rapidly switch between scanning for analytes’ masses (MS scan) and generating fragmentation spectra (MS/MS scan) of the ions detected and selected as parent ion in the MS scan. Linear ion traps, which have higher scan ranges, larger electronic trap fields, and higher resolution than quadrupole ion traps, were developed and succesfuly applied as extremely sensitive MS instruments concentrating ions in the trapping field for varying lengths of time [58].

FTICR MS uses high magnetic fields (7T) to trap the ions and cyclotron resonance to excite and detect the ions, with a resolution of >1,000,000 (separate *m*/*z* 1000.000 from *m*/*z* 1000.001). The advantages of an ultra-high magnetic field, such as 14.5T or 21 T, offers significant enhancement in overall performance of FTICR MS [59].

The development of tandem (MS/MS) instruments by selecting specific ions induces fragmentation and measures the m/z of the fragment ions was an important aspect of hybrid MALDI mass spectrometry. Further, we will discuss some applications of MALDI-MS/MS.

## 4. Applications of MALDI-MS/MS-Based Proteomics

Although most tandem mass spectrometry-based proteomic studies in biomedical research use ESI as an ionization source [60], MALDI-ToF/ToF tandem mass spectrometry remains a relatively simple and fast alternative to perform qualitative and quantitative analysis of amino and organic acids [6], proteins and peptides [61], protein–protein interactions (PPIs), and post-translational modifications (PTMs). A variety of peptides and proteins become biomarkers for clinical applications due to useful features, such as user-friendly sample preparation [62], preservation of non-covalent interactions, and high sensitivity, high-throughput screening procedures/fast data acquisition [63]. 

MALDI-MS/MS and MSI based on MS/MS are applicable from animal models to 3D cell cultures, from *Macaca mulatta*, one of the most utilized non-human primate species in biomedical research analyzed for cytosolic protein fraction from brain [64] to rat (*Rattus norvegicus*) for proteomic study of rat brain tissue FF and FFPE tissue sections [29] and sea urchin (*Strongylocentrotus purpuratus*) analyzed for proteotyping of egg membrane proteins [65]. Proteomic analysis by MALDI tandem mass spectrometry helps in deep understanding of various mechanisms involved in biological processes in living organisms, such as energy and metabolic processes [66,67,68]; clathrin-dependent endocytosis [69], defense against pathogens [66,70], and immune response [71]; cell communication, proliferation, and cell differentiation [72]; cell repair [70]; sperm motility [67]; capacitation, acrosome reaction and sperm-egg recognition [72]; protein turnover, protein folding, and stress response [67,68]; apoptotic process [73]; cytoskeleton organization [68]. 

A typical bottom-up proteomics workflow includes the following steps: isolation of the protein mixture from the biological sample, fractionation of proteins by gel electrophoresis or LC methods, trypsin cleavage of proteins, mass spectrometric measurements of the resulting peptides, and database search for protein identification [74]. In-tissue proteomics workflow based on tandem MALDI-MSI as well as MALDI MS/MS proteomics workflow for tissue homogenates/cell lysates and biofluids analysis are described below. 

### 4.1. Applications of MALDI-MS/MS for Solid Tissues Proteomics

#### 4.1.1. Applications of MALDI-MS/MS for In-Tissue Proteomics

**Figure 4:**
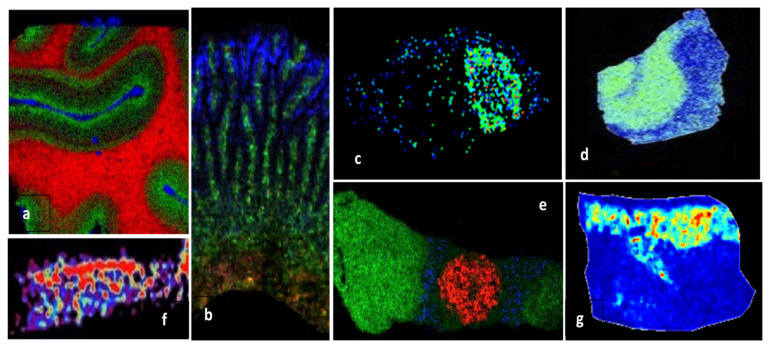
MALDI-MSI molecular pictures obtained by MALDI tandem mass spectrometry imaging for in situ identification of proteins: (**a**) Human visual cortex, myelin basic protein (red), neuromodulin (green), and hemoglobinβ (blue), MALDI-LTQ-Orbitrap instrument [75]; (**b**) rat small intestine, endogenous protein biomarkers in *lamina propria* (green), epithelium (blue), and submucosal layer (red), MALDI-ToF/ToF instrument [78]; (**c**) mouse model glioblastoma 60S ribosomal protein L34, MALDI-ToF/ToF instruments [83]; (**d**) AD hippocampal section, MUC19 isoform 5 (green), MALDI-ToF/TOF instrument [76]; (**e**) mouse pituitary gland, anterior lobe (green), vasopressin (red), γ-MSH (blue), MALDI-LTQ-Orbitrap [82]; (**f**) human articular cartilage, fibronectin distribution [77]; (**g**) Libechov minipig skin, normal skin, MALDI-ToF/ToF [81]. Reprinted and adapted with permission from Neagu A.-N., 2019. Proteome Imaging: [28].

MALDI MSI is a powerful label-free technique for mapping the spatial distribution of proteins in fresh frozen (FF) cryosections, as well as in formalin-fixed paraffin-embedded (FFPE) tissues, based on their tryptic fragments after on-tissue trypsinization. MSI, often based on MALDI tandem mass spectrometry, was successfully applied for proteomic analysis of various tissues (Table 2), such as post-mortem human brain samples (Figure 4a,d) [75,76], human articular cartilage [77] (Figure 4f), rats intestine [78] (Figure 4b), human atherosclerotic carotid [79], rat brain [29,80], normal and melanoma pig skin [81] (Figure 4g), mouse pituitary gland (Figure 4e) [82], or mouse model glioblastoma [83] (Figure 4c). 

MALDI-ToF/ToF was used in a MALDI-MSI proteomic study to identify and localize the dysregulated proteins and peptides within the mice testis sections, suggesting that male infertility is associated with loss of proteomic heterogeneity reflected in disruption of normal processes involved in spermatogenesis [85]. MALDI-ToF/ToF MSI analysis revealed spatially correlated lipid and protein changes in mouse heart [86] and mitochondrial and sarcomeric proteins from regions of interest (ROI) for identification of proteins/biomarkers in acute myocardial infraction (MI) tissue in human [84]. 

During complex workflows based on complementary methods for accurate identification of proteins/peptides, ultra-high mass resolution and accurate mass data were provided by MALDI-FT-ICR MSI of proteins that can be compared with the results obtained by MALDI-ToF/ToF MSI of in situ proteins and LC-ESI-MS/MS of protein extracts [83].

**Figure 5:**
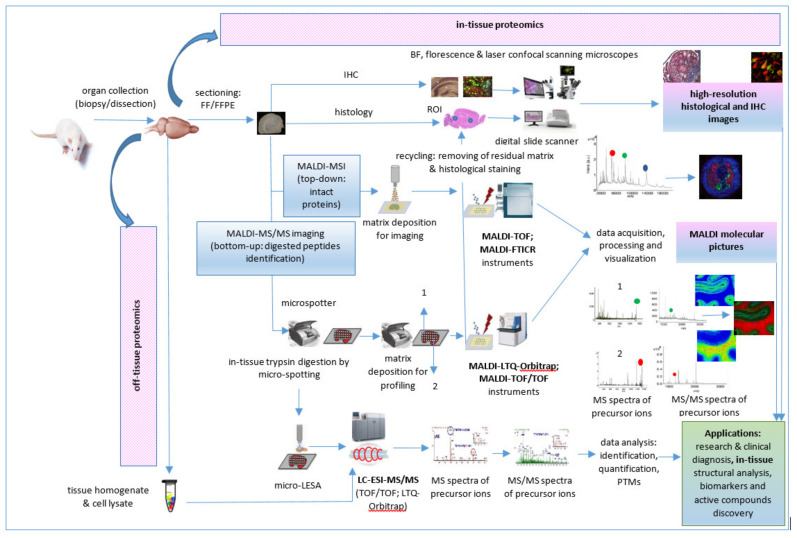
In-tissue proteomics workflow based on direct and tandem MALDI-MSI.

The first step into a MALDI MSI proteomic workflow (Figure 5) is sample acquisition/collection, handling, processing, and storage by dissection immediately after euthanasia of a model animal, autopsy, or during surgical procedures. Sample preparation follows sample collection. Usually, the tissue samples are cut on the cryostat, and the cuts of tissue are placed on indium tin oxide (ITO)-coated conductive glass slides, followed by the preparation of cryosections for on-tissue trypsinization and MALDI matrix application. Whole tissue trypsin digestion or automatic trypsin digestion using a microspotter device, such as a high accurate position chemical inkjet printer [29], can be followed by on-tissue derivatization [80]. After digestion, α-cyano-4-hydroxycinnamic acid (CHCA)/aniline (ANI) or other matrices are deposited on the same positions, also using the microspotter device. For MALDI-MS tandem MSI, the most used are a ToF/ToF instrument [83,87] and MALDI-linear quadrupole ion trap (LTQ)-Orbitrap hybrid mass spectrometer [75,82] operated in positive ion mode. Data acquisition, processing, and visualization is the last step of this proteomic workflow. Thus, the representative “molecular pictures” offer quantitative and spatial information about proteins without labeling of potential targets as in an immunohistochemistry (IHC) technique.

Often, IHC and haematoxylin and eosin (H & E) staining of tissue sections after MALDI-MSI are performed [87] to validate results and to correlate them with the microscopic-histopathological analysis of tissue samples. When performed by MALDI MSI bottom-up approach, a limited number of proteins can be identified directly at the tissue level [29]. Furthermore, the nanoLC-ESI-tandem mass spectrometry analysis can be performed for an efficient identification of proteins while keeping their spatial localization, after tissue micro-extraction using microscopy [76], liquid microjunction extraction/liquid extraction surface analysis (LESA) [29], or laser capture microdissection (LCM) followed by tissue/cell chemical or mechanical lysis, protein extraction, proteolytic digestions, and peptide purification for the removal of contaminants [83].

#### 4.1.2. Applications of MALDI-MS/MS for Off-Tissue Proteomics

**Figure 6:**
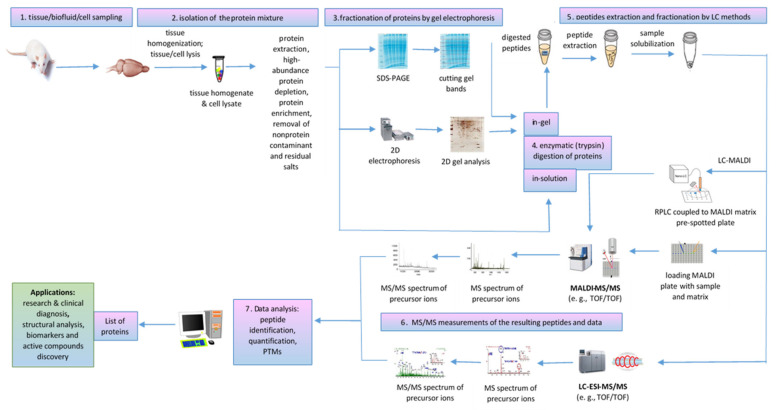
MALDI MS/MS proteomics workflow for tissue homogenates/cell lysates and biofluid analysis.

MALDI-ToF/ToF tandem mass spectrometry workflow (Figure 6) was successfully used for proteomic analysis of healthy and pathological tissues, homogenates, and lysates sampled from various organs by dissection or biopsy, such as senile plaques isolated from Alzheimer’s disease (AD) brain [88] and human frontal cortex associated with aging and age-related neurodegenerative diseases [89]; white muscles, for food authentication and identification of fish species of origin in processed products [90]; liver tissue samples obtained from HBV-infected mouse model, to provide novel insights into HBV-associated liver fibrosis [91]; retinal pigment epithelium (RPE), to detect light-induced phosphorylation of crystallins [92]; testis samples of azoospermia patients with Sertoli cell-only syndrome [73]; placenta, to compare the differentially expressed proteins in the fetal side compared to maternal side in spontaneous unexplained preterm labor with intact membrane (sPTL-IM) [71], etc. At cellular level, MALDI ToF/ToF can perform the detailed analysis of the proteome for a specific type of cell, from peripheral blood mononuclear cells (PBMCs) to identify novel biomarker candidates in rheumatoid arthritis [93] to spermatozoa proteomic analysis of carp [94] or asthenozoospermic [67] and normozoospermic infertile patients [72]. MALDI MS/MS is also useful for the organellar proteomic analysis of membrane proteins [95,96], mitochondrial proteome [97], rough endoplasmic reticulum (RER) [98], purified exosomes from human placenta [99] or from *Mycobacterium avium sp. paratuberculosis*-infected macrophages [100], nuclear pore complex [60], poultry egg white proteins [101], etc. Proteomic analysis of the airborne dust from school rooms by MALDI-ToF-MS and MALDI-ToF-MS/MS revealed that human epithelial cytokeratin 10 (CK10) is the most abundant protein, which confirmed that human skin is a source of large particles and their associated bacterial cells in household dust [102]. A proteomic protocol based on MALDI-ToF/ToF-based hydroxyproline mapping of collagen II (COLII), the most abundant protein in vertebrates that assures the normal structure and function of cartilage, showed that COLII can be isolated from *Capra hircus* ear cartilage and can be considered as an early biomarker of cartilage disorder as well as a possible constituent of an injectable hydrogel formulation that facilitates the differentiation of chondrocytes during the cartilage regeneration process [103] (Table 3). 

### 4.2. Applications of MALDI-MS/MS in Biofluids Proteomics

The recent development of various “omics” fields induced significant advances in the search for non-invasive biomarkers based on the analysis of body fluids for a wide spectrum of diseases, including malignancies [104]. MALDI-ToF/ToF tandem mass spectrometry was used or may be used in clinical settings and biomedical research as an analytical tool to investigate the protein expression profile within different biofluids, such as blood/serum/plasma, to identify the differentially expressed panels of proteins between patients and healthy controls in ovarian cancer (OC) [105] and breast cancer (BC) [106] or to detect proteins that can be used as biomarkers by comparing the levels found for patients with healthy controls, such as human osteopontin (OPN) [107]; urine, for microbial pathogen identification [108] or for characterization of proteomic patterns in gestational trophoblastic disease [109]; human reflex tear fluid, to provide new insights into the physiological function of human tears [110]; aqueous humor, for identification of a specific protein profile in cataract patients with pseudo-exfoliation [111]; saliva, for rapid screening to differentiate oral diseases from others pathologies [112], identification of putative biomarkers for orthodontic tooth movement [113] and changes in mouse whole saliva soluble proteome induced by tannin-enriched diet [114]; sputum, for investigation of tuberculosis pathogenesis and to discover biomarkers for detection of active pulmonary tuberculosis infection [115]; nasal fluid, for identification of antimicrobial peptide fingerprints [116]; human milk, to study casein phosphoproteome [117]; human cervicovaginal fluid (CVF), for identification of protein biomarkers for cervical cancer [118]; menstrual blood proteome, which may be used as a diagnostic tool for infertility and uterine pathologies or to aid in distinguishing menstrual blood from circulating blood in forensic [119]; human endometrial fluid aspirate, for better understanding endometriosis, endometrial cancer, and embryo implantation [120]; human follicular fluid (HFF), to better understand folliculogenesis and oocyte maturation and to discover biomarkers of female infertility and in vitro fertilization (IVF) outcome [121]; human cerebrospinal fluid (CSF) in post-traumatic condition of traumatic brain injury [122] or to analyze immunoaffinity depleted CSF and compare it with a non-depleted sample [123]; synovial fluid, for increasing knowledge on the etiopathogenesis of rheumatoid arthritis and osteoarthritis [124] and for investigation of citrullinated autoantigens as targets of anti-citrullinated protein antibodies response [125]; seminal plasma (SP) of turkey (*Meleagris gallopavo*) [66], Santa Ines Sheep [126], and carp [127], to improve the reproduction; pancreatic juice, for identification of overexpressed proteins from pancreatic ductal adenocarcinoma patients (PDAC) [128] and acute pancreatitis, to characterize pancreatic tissue damage [129]; exhaled breath condensate (EBC), to determine the EBC peptidome and to search for potential biomarkers for lung cancer diagnosis [130]; venom proteome of honeybee analyzed by hyphenated LC-MALDI ToF/ToF and LC-ESI QTOF approach, for identification of toxins, allergens, and bioactive pharmaceutical compounds [131] or from red-headed krait (*Bungarus flaviceps*), a venomous elapid snake, for understanding of snake venom molecular evolution and to contribute to effective treatment of krait bites [132]; oral cavity mucus in female tilapia fish (*Oreochromis spp.*), for understanding the functional aspects of mouthbrooding [68]; and epidermal mucus secretion of a cichlid, the discus fish (*Symphysodon aequifasciata*), to demonstrate parental-care behavior [70]. MALDI-Q-TOF tandem mass spectrometry was used to identify human skin keratins as the major proteins in EBC that derive from ambient air ant, not from the respiratory tract [133], as well as for proteins profiling the human malignant pleural effusions [134] (Table 4).

### 4.3. MALDI Tandem Mass Spectrometry Applications in Microbial Proteomics-Based Analyses

For bacterial identification, predominant MS techniques are ESI-MS/MS and MALDI-ToF-MS [135]. LC-ESI-MS/MS is the main technology in microbiological research, unlike MALDI, which occupies the clinical microbiology laboratory market despite its low possibility to target specific protein biomarkers or to handle complex microbial samples [136]. MALDI-ToF/ToF MS, complementary to MALDI-ToF MS and LC-ESI-MS/MS techniques, is useful for characterization of protein patterns, protein biomarkers, and whole proteomes for pathogenic bacteria [19] as well as for identification of bacteria based on secondary protein peaks, improving the identification of foodborne pathogens, such as *Bacillus cereus*, *Listeria monocytogenes*, and *Micrococcus luteus* [137]. Furthermore, MALDI-ToF/ToF coupled with MALDI-MSI can assure the discrimination between isomeric compound in ion images [138]. Thus, MALDI-MSI, which reveals the spatial distribution of protein biomarkers, coupled with MALDI-ToF/ToF MS for analysis of selected highly intensive mass peaks of tryptic peptides, can map the infectious biomarkers directly on surface of fresh frozen (FF) tissue sections, e.g., those made through porcine lymph nodes and lungs after respiratory infection [87]. In metaproteomics, MALDI-ToF/ToF MS implementation was discussed as a possible technique for more precise biomarker identifications in detection of antimicrobial resistance (AMR) in foodborne pathogens [139]. MALDI-ToF/ToF was used as an analytical tool for the determination of bacteria in urine samples collected from patients with urinary tract infections [108]. Furthermore, MALDI-ToF/ToF was successfully used for identification of full and truncated proteins produced by pathogenic *Escherichia coli* strains [140]. MALDI-ToF/ToF MS could aid in the generation of accurate molecular formulas and structural information to rapidly discriminate bacterial function [141] as well as for identification of new viral evasion strategies and fundamental factors governing host-microbial interactions as has been demonstrated in HPV infection that alters vaginal microbiome via downregulation of host mucosal innate peptides used by *Lactobacilli* as amino acid sources [142].

### 4.4. MALDI Tandem Mass Spectrometry Applications in Proteoforms Analysis

The presence inside the cell of splicing variants, protein isoforms, or fusion proteins demanded a more complete analysis using MALDI-ToF/ToF instruments [143]. MALDI-ToF/ToF tandem mass spectrometry method was developed as a high-throughput approach for the relative quantitation of the isomerized forms of different proteins and peptides, such as α- and β-Asp7 isoforms of amyloid-β peptide [144], as well as for the identification of D-amino acids in biological active peptides and proteins [145]. MALDI-ToF/ToF detected a wide variety of PTMs with key roles in cellular functions, such as glycosylation [146,147], acetylation [148], phosphorylation [149], or sulfonation [143]. MALDI-Q-ToF MS also contributed to detection of phosphopeptides present in peptide mixtures [150]. Liquid chromatography (LC) coupled with MALDI-ToF/ToF workflow was used for the structural characterization of protein complexes and PPIs by detection of crosslinks within and between proteins [151]. 

### 4.5. Applications of MALDI Tandem Mass Spectrometry in Oncoproteomics and Neuroproteomics

In oncoproteomics, MALDI-ToF/ToF as well as MALDI-Q-ToF were successfully used in MALDI-MSI approaches, alone or in combination with MALDI-MS and LC-ESI-MS/MS, for tumor classification [152], to demarcate tumor and non-tumor tissue regions [153], for assessing the phenotypic intratumor heterogeneity [154], to detect serum proteomic patterns and to discover novel protein biomarkers [105], to characterize the overexpressed and downregulated proteins in tumor cells and their tumor microenvironment (TME), to study cellular processes and pathways involved in apoptosis and metastasis, to monitor the response of carcinoma cells to different treatments [155], and even for investigation of the reported extensive protein glycosylation pattern alteration in cancer [153]. Thus, MALDI-MS/MS was involved in protein profile-based characterization of chemotherapy-sensitive and chemotherapy-resistant ER+ invasive ductal carcinoma (IDC) tissue samples [156], in proteomic investigation of TME in fresh frozen samples of BC [157], or in oral squamous cell carcinoma (OSCC) laser capture microdissected (LCM) sample analysis using LC separation and an ESI-MALDI tandem MS system [158].

LC-MALDI-MS/MS and MALDI-MSI investigation was used to compare the molecular profiles of primary tumors and their metastases in regional lymph nodes to emphasize molecular heterogeneity of papillary thyroid cancer stored as formalin-fixed paraffin-embedded (FFPE) samples [159]. MALDI-MSI coupled with MALDI-ToF/ToF was also used to assess the molecular differentiation in both FFPE and fresh-frozen colon adenocarcinoma tissue samples [160]. Overexpressed in various tumors, including breast cancer, the epidermal growth factor receptor (EGFR) was detected using an immune tandem mass spectrometry (iMALDI) diagnostic assay based on MALDI-MS to assess the molecular weight of the epitope-containing peptides and MALDI-MS/MS to perform their amino acid sequence [161]. 2DE-MALDI-ToF/ToF analysis identified the overexpressed proteins in adenoma and parathyroid hyperplasia, most of them involved in mitochondrial activity, with applications as biomarkers in differentiation of parathyroid hyperplasia from adenoma [162]. 

In neuroproteomics, MALDI-TOF tandem MS was successfully used for the characterization of proteins by sequencing of corresponding peptides present in senile plaques from brains of AD patients, emphasizing the putative role of these protein species in aggregation or proliferation of senile plaques [88]. MALDI-ToF MS/MS was used as a part of complex proteomic and imaging approach to emphasize the aluminum binding to modified amyloid-β peptides with implications in pathogenesis of AD [163]. 

### 4.6. Applications of MALDI-MS/MS for Identification of Bioactive Peptides and Proteins

MALDI-ToF/ToF MS-based proteomic approach has been used for the identification of D-amino acids in bioactive peptides and proteins in skin secretions from oriental fire-bellied toad (*Bombina orientalis*) [145], neurotoxins in venom of annulated sea snake (*Hydrophis cyanocinctus*) [164], cell-to-cell signaling neuropeptides in *Aplysia californica* neurons isolated from cerebral ganglia [165], as well as to discover novel neuropeptides of single neurohemal organ of flies that can aid in the development of neuropeptide-based control of these insects pests [166]. 

MALDI-ToF/ToF MS also contributed to analysis of the hyperglycemic hormone-family neuropeptides involved in the regulation of many physiological processes in crustaceans [167]. Honeybee (*Apis mellifera*) venom was analyzed through MALDI-ToF/ToF MS for detection and characterization of melittin, the main toxic peptide with membrane-disruptive abilities that can induce cancer cell death in aggressive triple-negative and HER2-enriched breast cancer subtypes [168], and apamin, which regulates gene expression in signaling pathways involved in cell development [169,170]. 

Additionally, MALDI-quadrupole ion trap time-of-flight (MALDI-QIT-ToF) MS was used for identification and characterization of disintegrins in *Crotalus horridus* snake venom [171], these cysteine-rich proteins emphasizing a role in cancer treatment due to their interaction with integrins involved in tumorigenesis, tumor growth, angiogenesis, and invasion/migration [172]. On-tissue MALDI-ToF/ToF fragmentation was carried out for confirmation of MALDI MSI peaks of neurotransmitters and small molecules in mouse and rat brain tissues [173]. The accurate mass and the amino acid sequence of a purified peptide were detected by MALDI-ToF/ToF MS, leading to characterization of a gloverin-like antimicrobial peptide isolated from muga silkworm (*Antheraea assamensis*) haemolymph after *Candida albicans* in vitro infection; the discovered antimicrobial peptides inhibited biofilm formation of the Gram-negative bacterial pathogens [174]. MALDI-ToF MS/MS analysis confirmed the presence and the role of porin protein in enhancement of antibacterial activity by epigenetic activation of an endophytic *Streptomyces coelicolor* strain that was isolated from the radicular surface of neem plant (*Azadirachta indica*) [175]. 

### 4.7. Application of MALDI-MS/MS-Based Proteomics in Exposomics and Foodomics

MALDI-ToF/ToF-MS and LC-Q-ToF-MS were used to explore a wide spectrum of proteinaceous adducts as putative biomarkers for retrospective exposure to environmental pollutants, agents for bioterrorism and warfare, such as organophosphate and organophosphate ester adducts of albumin [176], and sulphur mustard adducts of hemoglobin, metallothioneins (MTs), and cysteine-rich and heavy metal-binding proteins [177,178]. MALDI-ToF/ToF also detected the protein toxins, such as botulinum neurotoxins, *Clostridium perfringens* epsilon toxin, staphylococcal enterotoxin B, Shiga toxin (Stx), and plant toxin ricin [179]. Additionally, MALDI-ToF/ToF MS is an important tool for proteomic analysis of food allergens from milk, egg, hazelnut, and lupin seeds [180]; fish, such as herring [181] and rainbow trout (*Oncorhynchus mykiss*) [182]; or even crocodile meat that can be a severe food allergen [183]. A hybrid MALDI Q-ToF mass spectrometer was used for identification of the proteoform profiles to detect cow, goat, sheep, and camel milk adulteration for assessing food quality [184]. 

MALDI-ToF/ToF technique identified some antidiabetic-related proteins from grey oyster mushroom (*Pleurotus pulmonarius*) basidiocarps, involved in decreasing insulin resistance and vascular complications in type-2 diabetes mellitus [185]. MALDI-ToF/ToF analysis revealed the presence of highly abundant proteins with possible bio-efficacy in medicinal mushroom *Ganoderma lucidum*, commonly used in traditional Chinese medicine [186]. A nanoLC-MALDI-ToF/ToF MS system analyzed the proteomic composition of royal jelly (RJ) that can contribute to the development of standards and regulations, quality enhancement, and safety of this essential dietary supplement produced by honeybees [187].

In exposomics, MALDI ToF-MS/MS identified proteins that are involved in oxidative stress/redox status, cytoprotection, lysosomal degradation, immune response, signal transduction, ionoregulation, metabolic pathways, protein modifications, autophagy, and clathrin-mediated endocytosis, as putative biomarkers for aquatic systems monitoring based on different sentinel species, such as medaka (*Oryzias melastigma*) exposed to acute inorganic mercury as persistent pollutant [188] and mussel (*Mytilus galloprovincialis*) exposed to silver nanoparticles toxicity [69]. 

### 4.8. Applications of MALDI Tandem Mass Spectrometry in Other Domains

Identification of proteins by MALDI-ToF MS can be followed by MALDI-ToF/ToF in order to analyze the proteinaceous binders in historical painting samples in cultural heritage [189]. Additionally, in paleoproteomics or zooarcheology based on MS, MALDI-MS was used to detect proteins, such as osteocalcin, from fossil tissue of various extinct species, for subsequent analysis of the protein’s/peptide’s primary sequence using MALDI-MS/MS [190]. 

## 5. Advantages and Limitations

In terms of the advantages and limitations of MALDI ToF technology, we can say that the limitations are probably insignificant in terms of researchers’ progress toward putting MALDI into the clinic. The advantage of the MALDI-TOF in microbiological diagnostics is its low supply cost, technician processing time (ten minutes), and overall 95% accuracy at the species level, allowing for faster and more accurate treatment for patients. Furthermore, because ions have low internal energy, the use of “soft” ionization in MALDI-ToF allows for the observation of ionized molecules with little to no fragmentation, providing direct molecular weight assessment. The greatest limitations of MALDI-ToF include low analytical sensitivity without prior culture and the inability to distinguish between related species, which may be due to the organisms’ inherent similarities. As a result, MALDI-ToF is not a tool that can detect a low amount of bacteria that may be present in sterile samples, such as cerebrospinal fluids, and more standardization and optimization will be required. The practice of clinical microbiology will be transformed as these platforms improve and become more widely available.

Using multimodal platform of mass spectrometry and imaging in combination with other methods, such as histochemistry, immunohistochemistry, and data science, MALDI will be extensively used in clinics to evaluate drugs’ efficacy, their impact on protein biomarkers, the molecular distribution on tissues, and microbial monitoring. The potential of MALDI mass spectrometric analysis for tissue imaging is high, and advancements in instrumentation and processing approaches can offer new developments for structural elucidation of biomolecules related to health and disease.

## 6. Conclusions

In this review, we discussed the principles of MALDI tandem mass spectrometry, including a detailed proteomics experimental design for on-tissue mapping, identification and characterization of proteins and peptides by MALDI-MSI, as well as the proteomics workflow for MALDI-MS/MS applied to liquid biological samples, including tissue/cell lysates and biofluids. MALDI-MS/MS technique, especially based on ToF/ToF instruments, is complementary to LC-ESI-MS/MS methods, with applications in oncoproteomics, neuroproteomics, exposomics, foodomics, microbiology-based proteomics analysis, identification of novel biomarkers, and new biological active molecules. Tandem mass spectrometry techniques allow the transition to an untargeted proteomics approach, sustaining the identification of PTMs and PPIs and emphasizing their molecular functions and implication in biological processes in normal and diseased tissues and cells in humans, animal models, bacteria, and other living systems, such as cell culture and complex communities from environmental samples.

## Figures and Tables

**Table 1:** Various Application of Organic Matrices in MALDI-MS.

Matrix	Application	References
ɑ-cyano-4-hydroxycinnamic acid (CHCA)	small molecules, peptides/proteins < 6 kDa	[40,41,42]
2,5-dihydroxybenzoic acid (DHB)	small molecules, peptides/proteins < 6 kDa, polymers, carbohydrates	[40,41,42]
α-cyano-5-phenyl-2,4-pentadienic acid (CPPA)	proteins	[43,44]
3,5-dimethoxy-4-hydroxycinnamic acid (SA, sinapinic acid)	proteins	[43,44]
2-(4-Hydroxyphenylazo)benzoic acid (HABA)	peptides, proteins, glycoproteins	[45]
9-aminoacridine (9-AA)	small molecules, lipids, MALDI (−)	[46]

**Table 2:** In-tissue proteomic analysis of different normal and diseased tissues by MALDI MSI based on MS/MS technique.

Tissue/Organ	References
human brain	[75,76]
human acute myocardial infraction tissue	[84]
human articular cartilage	[77]
human atherosclerotic carotid	[79]
rat brain	[29,80]
rat intestine	[78]
pig skin (normal and melanoma)	[81]
mouse model glioblastoma	[83]
mouse testis	[85]
mouse heart	[86]
mouse pituitary gland	[82]

**Table 3:** Applications of MALDI-MS/MS for off-tissue proteomics.

Organ/Tissue/Cell Homogenates and Cell Lysates	References
senile plaques from AD brain	[88]
human frontal cortex	[89]
human testis	[73]
human placenta	[71]
fish white muscles	[90]
mouse model liver fibrosis	[91]
retinal pigment epithelium (RPE)	[92]
mononuclear cells	[93]
spermatozoa	[72,94]
plasma membrane	[95,96]
mitochondria	[97]
rough endoplasmic reticulum (RER)	[98]
exosomes	[99,100]
nuclear pore complex	[60]
egg white	[101]
cytokeratins in household dust	[102]
collagens from cartilage	[103]

**Table 4:** Biofluids proteomic analysis using MALDI tandem mass spectrometry.

Biofluids	References
blood/serum/plasma	[105,106]
urine	[109]
human reflex tear fluid	[110]
aqueous humor	[111]
saliva	[112,113,114]
sputum	[115]
nasal fluid	[116]
human milk	[117]
human cervicovaginal fluid (CVF)	[118]
menstrual blood	[119]
human endometrial fluid aspirate	[120]
human follicular fluid (HFF)	[121]
human cerebrospinal fluid (CSF)	[122,123]
synovial fluid	[124,125]
seminal plasma	[66,126,127]
pancreatic juice	[128,129]
exhaled breath condensate (EBC)	[130]
venom	[131,132]
oral cavity mucus	[68]
epidermal mucus	[70]
human malignant pleural effusions	[134]

## Data Availability

Not applicable.

## References

[1] Karas M., Bachmann D., Bahr U., Hillenkamp F. (1987). Matrix-assisted ultraviolet laser desorption of non-volatile compounds. Int. J. Mass Spectrom. Ion Processes.

[2] Wysocki V.H., Resing K.A., Zhang Q.F., Cheng G.L. (2005). Mass spectrometry of peptides and proteins. Methods.

[3] Tabet J.C., Rebuffat S. (2003). Nobel Prize 2002 for chemistry: Mass spectrometry and nuclear magnetic resonance. M S-Med. Sci..

[4] Brodbelt J.S., Reid G.E. (2020). Special Focus: Honoring John Yates for Receiving the 2019 John B. Fenn Award for a Distinguished Contribution in Mass Spectrometry. J. Am. Soc. Mass Spectr..

[5] Nadler W.M., Waidelich D., Kerner A., Hanke S., Berg R., Trumpp A., Rösli C. (2017). MALDI versus ESI: The Impact of the Ion Source on Peptide Identification. J. Proteome Res..

[6] Gogichaeva N.V., Williams T., Alterman M.A. (2007). MALDI TOF/TOF Tandem Mass Spectrometry as a New Tool for Amino Acid Analysis. J. Am. Soc. Mass Spectr..

[7] Gu H., Ma K., Zhao W., Qiu L., Xu W. (2021). A general purpose MALDI matrix for the analyses of small organic, peptide and protein molecules. Analyst.

[8] Herkt M., Foinquinos A., Batkai S., Thum T., Pich A. (2020). Pharmacokinetic Studies of Antisense Oligonucleotides Using MALDI-TOF Mass Spectrometry. Front. Pharm..

[9] Wang J., Zhao J., Nie S., Xie M., Li S. (2022). Rapid profiling strategy for oligosaccharides and polysaccharides by MALDI TOF mass spectrometry. Food Hydrocoll..

[10] Fresnais M., Yildirim E., Karabulut S., Jäger D., Zörnig I., Benzel J., Pajtler K.W., Pfister S.M., Burhenne J., Haefeli W.E. (2021). Rapid MALDI-MS Assays for Drug Quantification in Biological Matrices: Lessons Learned, New Developments, and Future Perspectives. Molecules.

[11] Lee J.H., Kim Y.H., Kim K.-H., Cho J.Y., Woo S.M., Yoo B.C., Kim S.C. (2018). Profiling of Serum Metabolites Using MALDI-TOF and Triple-TOF Mass Spectrometry to Develop a Screen for Ovarian Cancer. Cancer Res. Treat.

[12] Wang Z., Zhang Q., Shen H., Yang P., Zhou X. (2021). Optimized MALDI-TOF MS Strategy for Characterizing Polymers. Front. Chem..

[13] Glocker M.O., Bauer S.H.J., Kast J., Volz J., Przybylski M. (1996). Characterization of specific noncovalent protein complexes by UV matrix-assisted laser desorption ionization mass spectrometry. J. Mass Spectrom..

[14] Singhal N., Kumar M., Kanaujia P.K., Virdi J.S. (2015). MALDI-TOF mass spectrometry: An emerging technology for microbial identification and diagnosis. Front. Microbiol..

[15] El-Aneed A., Cohen A., Banoub J. (2009). Mass Spectrometry, Review of the Basics: Electrospray, MALDI, and Commonly Used Mass Analyzers. Appl. Spectrosc. Rev..

[16] Gobey J., Cole M., Janiszewski J., Covey T., Chau T., Kovarik P., Corr J. (2005). Characterization and Performance of MALDI on a Triple Quadrupole Mass Spectrometer for Analysis and Quantification of Small Molecules. Anal. Chem..

[17] Magparangalan D.P., Garrett T.J., Drexler D.M., Yost R.A. (2010). Analysis of Large Peptides by MALDI Using a Linear Quadrupole Ion Trap with Mass Range Extension. Anal. Chem..

[18] Fox A. (2006). Mass spectrometry for species or strain identification after culture or without culture: Past, present, and future. J. Clin. Microbiol..

[19] Intelicato-Young J., Fox A. (2013). Mass spectrometry and tandem mass spectrometry characterization of protein patterns, protein markers and whole proteomes for pathogenic bacteria. J. Microbiol. Methods.

[20] Nakashima Y., Nahar S., Miyagi-Shiohira C., Kinjo T., Toyoda Z., Kobayashi N., Saitoh I., Watanabe M., Fujita J., Noguchi H. (2018). A Liquid Chromatography with Tandem Mass Spectrometry-Based Proteomic Analysis of the Proteins Secreted by Human Adipose-Derived Mesenchymal Stem Cells. Cell Transpl..

[21] Neagu A.-N., Jayathirtha M., Baxter E., Donnelly M., Petre B.A., Darie C.C. (2022). Applications of Tandem Mass Spectrometry (MS/MS) in Protein Analysis for Biomedical Research. Molecules.

[22] Fernández-Puente P., Mateos J., Blanco F.J., Ruiz-Romero C., Martins-de-Souza D. (2014). LC-MALDI-TOF/TOF for Shotgun Proteomics. Shotgun Proteomics: Methods and Protocols.

[23] Bashyal A., Sanders J.D., Holden D.D., Brodbelt J.S. (2019). Top-Down Analysis of Proteins in Low Charge States. J. Am. Soc. Mass Spectr..

[24] Campuzano I.D.G., Robinson J.H., Hui J.O., Shi S.D.H., Netirojjanakul C., Nshanian M., Egea P.F., Lippens J.L., Bagal D., Loo J.A. (2019). Native and Denaturing MS Protein Deconvolution for Biopharma: Monoclonal Antibodies and Antibody-Drug Conjugates to Polydisperse Membrane Proteins and Beyond. Anal. Chem..

[25] Hebert A.S., Prasad S., Belford M.W., Bailey D.J., McAlister G.C., Abbatiello S.E., Huguet R., Wouters E.R., Dunyach J.J., Brademan D.R. (2018). Comprehensive Single-Shot Proteomics with FAIMS on a Hybrid Orbitrap Mass Spectrometer. Anal. Chem..

[26] Griffiths R.L., Hughes J.W., Abbatiello S.E., Belford M.W., Styles I.B., Cooper H.J. (2020). Comprehensive LESA Mass Spectrometry Imaging of Intact Proteins by Integration of Cylindrical FAIMS. Anal. Chem..

[27] Spraggins J.M., Djambazova K.V., Rivera E.S., Migas L.G., Neumann E.K., Fuetterer A., Suetering J., Goedecke N., Ly A., Van de Plas R. (2019). High-Performance Molecular Imaging with MALDI Trapped Ion-Mobility Time-of-Flight (timsTOF) Mass Spectrometry. Anal. Chem..

[28] Neagu A.-N. (2019). Proteome Imaging: From Classic to Modern Mass Spectrometry-Based Molecular Histology. Advancements of Mass Spectrometry in Biomedical Research.

[29] Quanico J., Franck J., Dauly C., Strupat K., Dupuy J., Day R., Salzet M., Fournier I., Wisztorski M. (2012). Development of liquid microjunction extraction strategy for improving protein identification from tissue sections. J. Proteom..

[30] Miller R.M., Ibrahim K., Smith L.M. (2021). ProteaseGuru: A Tool for Protease Selection in Bottom-Up Proteomics. J. Proteome Res..

[31] Kertesz V., Cahill J.F. (2021). Spatially resolved absolute quantitation in thin tissue by mass spectrometry. Anal. Bioanal. Chem..

[32] Lamont L., Hadavi D., Viehmann B., Flinders B., Heeren R.M.A., Vreeken R.J., Porta Siegel T. (2021). Quantitative mass spectrometry imaging of drugs and metabolites: A multiplatform comparison. Anal. Bioanal. Chem..

[33] Koeniger S.L., Talaty N., Luo Y., Ready D., Voorbach M., Seifert T., Cepa S., Fagerland J.A., Bouska J., Buck W. (2011). A quantitation method for mass spectrometry imaging. Rapid Commun. Mass Spectrom. RCM.

[34] Vertes A., Irinyi G., Gijbels R. (1993). Hydrodynamic Model of Matrix-Assisted Laser-Desorption Mass-Spectrometry. Anal. Chem..

[35] Zenobi R., Knochenmuss R. (1998). Ion formation in MALDI mass spectrometry. Mass Spectrom. Rev..

[36] Batoy S.M.A.B., Akhmetova E., Miladinovic S., Smeal J., Wilkins C.L. (2008). Developments in MALDI mass spectrometry: The quest for the perfect matrix. Appl. Spectrosc. Rev..

[37] Knochenmuss R., Dubois F., Dale M.J., Zenobi R. (1996). The matrix suppression effect and ionization mechanisms in matrix-assisted laser desorption/ionization. Rapid Commun. Mass Sp..

[38] Karas M., Hillenkamp F. (1988). Laser Desorption Ionization of Proteins with Molecular Masses Exceeding 10000 Daltons. Anal. Chem..

[39] Tingting Tu M.L.G. (2019). Miniaturizing sample spots for matrix-assisted laser desorption/ionization mass spectrometry. Trends Anal. Chem.

[40] Leopold J., Popkova Y., Engel K.M., Schiller J. (2018). Recent Developments of Useful MALDI Matrices for the Mass Spectrometric Characterization of Lipids. Biomolecules.

[41] Wang Y.-H.L.a.Y.-S. (2017). Matrix-Assisted Laser Desorption/Ionization Mass Spectrometry: Mechanistic Studies and Methods for Improving the Structural Identification of Carbohydrates. Mass Spectrom..

[42] Guerrera I.C., Kleiner O. (2005). Application of mass spectrometry in proteomics. Biosci. Rep..

[43] Monopoli A., Nacci A., Cataldi T.R.I., Calvano C.D. (2020). Synthesis and Matrix Properties of alpha-Cyano-5-phenyl-2,4-pentadienic Acid (CPPA) for Intact Proteins Analysis by Matrix-Assisted Laser Desorption/Ionization Mass Spectrometry. Molecules.

[44] Cadene M., Chait B.T. (2000). A robust, detergent-friendly method for mass spectrometric analysis of integral membrane proteins. Anal. Chem..

[45] Clark A.E., Kaleta E.J., Arora A., Wolk D.M. (2013). Matrix-assisted laser desorption ionization-time of flight mass spectrometry: A fundamental shift in the routine practice of clinical microbiology. Clin. Microbiol. Rev..

[46] Vermillion-Salsbury R., Hercules D. (2002). General equation for calculating the dissociation constants of polyprotic acids and bases from measured retention factors in high-performance liquid chromatography. Rapid Commun. Mass Spectrom..

[47] Cramer R., Pirkl A., Hillenkamp F., Dreisewerd K. (2013). Liquid AP-UV-MALDI enables stable ion yields of multiply charged peptide and protein ions for sensitive analysis by mass spectrometry. Angew. Chem. Int. Ed. Engl..

[48] Ryumin P., Brown J., Morris M., Cramer R. (2016). Investigation and optimization of parameters affecting the multiply charged ion yield in AP-MALDI MS. Methods.

[49] Piras C., Ceniti C., Hartmane E., Costanzo N., Morittu V.M., Roncada P., Britti D., Cramer R. (2020). Rapid Liquid AP-MALDI MS Profiling of Lipids and Proteins from Goat and Sheep Milk for Speciation and Colostrum Analysis. Proteomes.

[50] Siuzdak G. (1996). Mass Spectrometry for Biotechnology.

[51] Guilhaus M., Selby D., Mlynski V. (2000). Orthogonal acceleration time-of-flight mass spectrometry. Mass Spectrom. Rev..

[52] Zubarev R.A., Makarov A. (2013). Orbitrap Mass Spectrometry. Anal. Chem..

[53] Liu X.R., Zhang M.M., Gross M.L. (2022). Mass Spectrometry-Based Protein Footprinting for High Order Structure Analysis: Fundamentals and Applications. Chem. Rev..

[54] Chen Y., Leach F.E., Kaiser N.K., Dang X., Ibrahim Y.M., Norheim R.V., Gordon A.A., Richard D.S., Alan G.M. (2015). Improved ion optics for introduction of ions into a 9.4-T Fourier transform ion cyclotron resonance mass spectrometer. J. Mass Spectrom..

[55] Chernushevich I.V., Loboda A.V., Thomson B.A. (2001). An introduction to quadrupole-time-of-flight mass spectrometry. J. Mass Spectrom..

[56] Petre B.A., Youhnovski N., Lukkari J., Weber R., Przybylski M. (2005). Structural Characterisation of tyrosine-nitrated peptides by ultraviolet and infrared matrix-assisted laser desorption/ionization Fourier transforms ion cyclotron resonance mass spectrometry. Eur. J. Mass Spectrom..

[57] March R.E. (2000). Quadrupole ion trap mass spectrometry: A view at the turn of the century. Int. J. Mass Spectrom..

[58] Lu I.C., Lin J.L., Lai S.-H., Chen C.-H. (2011). Frequency-Scanning MALDI Linear Ion Trap Mass Spectrometer for Large Biomolecular Ion Detection. Anal. Chem..

[59] Marshall A.G., Hendrickson C.L., Emmett M.R., Rodgers R.P., Blakney G.T., Nilsson C.L. (2007). Fourier transform ion cyclotron resonance: State of the art. Eur. J. Mass Spectrom..

[60] Huang L., Baldwin M., Maltby D., Medzihradszky K., Baker P., Allen N., Rexach M., Edmondson R., Campbell J., Juhasz P. (2002). The Identification of Protein-Protein Interactions of the Nuclear Pore Complex of Saccharomyces cerevisiae Using High Throughput Matrix-assisted Laser Desorption Ionization Time-of-Flight Tandem Mass Spectrometry. Mol. Cell. Proteom. MCP.

[61] Santacruz C.P., Ayala E., Costa-Vera C. (2006). Design and performance of a matrix-assisted laser desorption/ionization tandem time-of-flight mass spectrometer. Braz. J. Phys..

[62] Dave K., Headlam M., Wallis T., Gorman J. (2011). Preparation and analysis of proteins and peptides using MALDI TOF/TOF mass spectrometry. Curr. Protoc. Protein Sci..

[63] Giampà M., Sgobba E. (2020). Insight to Functional Conformation and Noncovalent Interactions of Protein-Protein Assembly Using MALDI Mass Spectrometry. Molecules.

[64] Tannu N.S., Hemby S.E. (2007). De novo protein sequence analysis of Macaca mulatta. BMC Genom..

[65] Yergey A.L., Coorssen J.R., Backlund P.S., Blank P.S., Humphrey G.A., Zimmerberg J., Campbell J.M., Vestal M.L. (2002). De novo sequencing of peptides using MALDI/TOF-TOF. J. Am. Soc. Mass Spectr..

[66] Slowinska M., Nynca J., Arnold G.J., Fröhlich T., Jankowski J., Kozłowski K., Mostek A. (2017). Proteomic identification of turkey (Meleagris gallopavo) seminal plasma proteins1,2. Poult. Sci..

[67] Siva A.B., Kameshwari D.B., Singh V., Pavani K., Sundaram C.S., Rangaraj N., Deenadayal M., Shivaji S. (2010). Proteomics-based study on asthenozoospermia: Differential expression of proteasome alpha complex. Mol. Hum. Reprod..

[68] Iq K.C., Shu-Chien A.C. (2011). Proteomics of buccal cavity mucus in female tilapia fish (*Oreochromis* spp.): A comparison between parental and non-parental fish. PLoS ONE.

[69] Bouallegui Y., Ben Younes R., Oueslati R., Sheehan D. (2018). Redox proteomic insights into involvement of clathrin-mediated endocytosis in silver nanoparticles toxicity to Mytilus galloprovincialis. PLoS ONE.

[70] Chong K., Joshi S., Jin L., Shu-Chien A. (2006). Proteomics profiling of epidermal mucus secretion of a cichlid (Symphysodon aequifasciata) demonstrating parental care behavior. Proteomics.

[71] Tan N., Daim L., Mohd Jamil A., Mohtarrudin N., Karuppiah T. (2018). Spontaneous Unexplained Preterm Labor with Intact Membrane: Finding Protein Biomarkers through Placenta Proteome.

[72] Xu W., Hu H., Wang Z., Chen X., Yang F., Zhu Z., Fang P., Dai J., Wang L., Shi H. (2012). Proteomic characteristics of spermatozoa in normozoospermic patients with infertility. J. Proteom..

[73] Li J., Guo W., Li F., He J., Yu Q., Wu X., Li J., Mao X. (2012). HnRNPL as a key factor in spermatogenesis: Lesson from functional proteomic studies of azoospermia patients with sertoli cell only syndrome. J. Proteom..

[74] Dupree E.J., Jayathirtha M., Yorkey H., Mihasan M., Petre B.A., Darie C.C. (2020). A Critical Review of Bottom-Up Proteomics: The Good, the Bad, and the Future of this Field. Proteomes.

[75] González de San Román E., Bidmon H.-J., Malisic M., Susnea I., Küppers A., Hübbers R., Wree A., Nischwitz V., Amunts K., Huesgen P.F. (2018). Molecular composition of the human primary visual cortex profiled by multimodal mass spectrometry imaging. Brain Struct. Funct..

[76] Ho Kim J., Franck J., Kang T., Heinsen H., Ravid R., Ferrer I., Hee Cheon M., Lee J.-Y., Shin Yoo J., Steinbusch H.W. (2015). Proteome-wide characterization of signalling interactions in the hippocampal CA4/DG subfield of patients with Alzheimer’s disease. Sci. Rep..

[77] Rocha B., Cillero-Pastor B., Blanco F.J., Ruiz-Romero C. (2017). MALDI mass spectrometry imaging in rheumatic diseases. Biochim. Et Biophys. Acta (BBA)-Proteins Proteom..

[78] Nilsson A., Peric A., Strimfors M., Goodwin R.J.A., Hayes M.A., Andrén P.E., Hilgendorf C. (2017). Mass Spectrometry Imaging proves differential absorption profiles of well-characterised permeability markers along the crypt-villus axis. Sci. Rep..

[79] Martin-Lorenzo M., Balluff B., Sanz-Maroto A., van Zeijl R.J.M., Vivanco F., Alvarez-Llamas G., McDonnell L.A. (2014). 30μm spatial resolution protein MALDI MSI: In-depth comparison of five sample preparation protocols applied to human healthy and atherosclerotic arteries. J. Proteom..

[80] Franck J., el Ayed M., Wisztorski M., Salzet M., Fournier I. (2010). On Tissue Protein Identification Improvement by N-Terminal Peptide Derivatization. Methods Mol. Biol..

[81] Guran R., Vanickova L., Horak V., Krizkova S., Michalek P., Heger Z., Zitka O., Adam V. (2017). MALDI MSI of MeLiM melanoma: Searching for differences in protein profiles. PLoS ONE.

[82] Schulz S., Römpp A., Kummer W., Spengler B. (2011). AP-MALDI Imaging of Neuropeptides in Mouse Pituitary Gland with 5 ??m Spatial Resolution and High Mass Accuracy.

[83] Dilillo M., Ait-Belkacem R., Esteve C., Pellegrini D., Nicolardi S., Costa M., Vannini E., Graaf E.L.d., Caleo M., McDonnell L.A. (2017). Ultra-High Mass Resolution MALDI Imaging Mass Spectrometry of Proteins and Metabolites in a Mouse Model of Glioblastoma. Sci. Rep..

[84] Yajima Y., Hiratsuka T., Kakimoto Y., Ogawa S., Shima K., Yamazaki Y., Yoshikawa K., Tamaki K., Tsuruyama T. (2018). Region of Interest analysis using mass spectrometry imaging of mitochondrial and sarcomeric proteins in acute cardiac infarction tissue. Sci. Rep..

[85] Lahiri S., Aftab W., Walenta L., Strauss L., Poutanen M., Mayerhofer A., Imhof A. (2021). MALDI-IMS combined with shotgun proteomics identify and localize new factors in male infertility. Life Sci. Alliance.

[86] Kaya I., Sämfors S., Levin M., Borén J., Fletcher J.S. (2020). Multimodal MALDI Imaging Mass Spectrometry Reveals Spatially Correlated Lipid and Protein Changes in Mouse Heart with Acute Myocardial Infarction. J. Am. Soc. Mass Spectr..

[87] Do T., GurâH R., Jarpaová R., Ondrac Kova P., Sládek Z., Faldyna M., Adam V.c., Zítka O. (2020). MALDI MSI Reveals the Spatial Distribution of Protein Markers in Tracheobronchial Lymph Nodes and Lung of Pigs after Respiratory Infection. Molecules.

[88] Kelley A., Perry G., Bach S. (2018). Characterization of Proteins Present in Isolated Senile Plaques from Alzheimer’s Diseased Brains by MALDI-TOF MS with MS/MS. ACS Chem. Neurosci..

[89] Pan S., Shi M., Jin J., Albin R., Lieberman A., Gearing M., Lin B., Pan C., Yan X., Kashima D.T. (2007). Proteomics Identification of Proteins in Human Cortex Using Multidimensional Separations and MALDI Tandem Mass Spectrometer.

[90] Barik S., Banerjee S., Bhattacharjee S., Das Gupta S., Mohanty S., Mohanty B. (2013). Proteomic Analysis of Sarcoplasmic Peptides of Two Related Fish Species for Food Authentication. Appl. Biochem. Biotechnol..

[91] Kan F., Ye L., Yan T., Cao J., Zheng J., Li W. (2017). Proteomic and transcriptomic studies of HBV-associated liver fibrosis of an AAV-HBV-infected mouse model. BMC Genom..

[92] Lee H., Chung H., Lee S.H., Jahng W.J. (2011). Light-Induced Phosphorylation of Crystallins in the Retinal Pigment Epithelium. Int. J. Biol. Macromol..

[93] Xinqiang S., Kaiming L., Lei C., Lim T.K., Lee Y.M., Yuan L. (2018). Quantitative proteomic analysis of peripheral blood mononuclear cells in rheumatoid arthritis. Rheumatol. Orthop. Med..

[94] Dietrich M.A., Ciereszko A. (2018). Proteomic characterization of fresh spermatozoa and supernatant after cryopreservation in relation to freezability of carp (*Cyprinus carpio* L) semen. PLoS ONE.

[95] Poetsch A., Schlüsener D., Florizone C., Eltis L., Menzel C., Rögner M., Steinert K., Roth U. (2008). Improved Identification of Membrane Proteins by MALDI-TOF MS/MS Using Vacuum Sublimated Matrix Spots on an Ultraphobic Chip Surface. J. Biomol. Tech. JBT.

[96] Meier-Credo J., Preiss L., Wüllenweber I., Resemann A., Nordmann C., Zabret J., Suckau D., Michel H., Nowaczyk M.M., Meier T. (2022). Top-Down Identification and Sequence Analysis of Small Membrane Proteins Using MALDI-MS/MS. J. Am. Soc. Mass Spectr..

[97] Alonso J., Rodriguez J.M., Baena-López L.A., Santarén J.F. (2005). Characterization of the Drosophila melanogaster Mitochondrial Proteome. J. Proteome Res..

[98] Chen X., Sans M.D., Strahler J.R., Karnovsky A., Ernst S.A., Michailidis G., Andrews P.C., Williams J.A. (2010). Quantitative Organellar Proteomics Analysis of Rough Endoplasmic Reticulum from Normal and Acute Pancreatitis Rat Pancreas. J. Proteome Res..

[99] Burkova E.E., Grigor’eva A.E., Bulgakov D.V., Dmitrenok P.S., Vlassov V.V., Ryabchikova E.I., Sedykh S.E., Nevinsky G.A. (2019). Extra Purified Exosomes from Human Placenta Contain an Unpredictable Small Number of Different Major Proteins. Int. J. Mol. Sci..

[100] Wang J.-j., Chen C., Xie P.-f., Pan Y., Tan Y.-h., Tang L.-j. (2014). Proteomic analysis and immune properties of exosomes released by macrophages infected with Mycobacterium avium. Microbes Infect..

[101] Hu S., Qiu N., Liu Y., Zhao H., Gao D., Song R., Ma M. (2016). Identification and comparative proteomic study of quail and duck egg white protein using 2-dimensional gel electrophoresis and matrix-assisted laser desorption/ionization time-of-flight tandem mass spectrometry analysis. Poult. Sci..

[102] Fox K., Castanha E., Fox A., Feigley C., Salzberg D. (2008). Human K10 epithelial keratin is the most abundant protein in airborne dust of both occupied and unoccupied school rooms. J. Environ. Monit. JEM.

[103] Maity P.P., Dutta D., Ganguly S., Kapat K., Dixit K., Chowdhury A.R., Samanta R., Das N.C., Datta P., Das A.K. (2019). Isolation and mass spectrometry based hydroxyproline mapping of type II collagen derived from Capra hircus ear cartilage. Commun. Biol..

[104] Li J., Guan X., Fan Z., Ching L.-M., Li Y., Wang X., Cao W.-M., Liu D.-X. (2020). Non-Invasive Biomarkers for Early Detection of Breast Cancer. Cancers.

[105] Swiatly A., Horala A., Hajduk J., Matysiak J., Nowak-Markwitz E., Kokot Z.J. (2017). MALDI-TOF-MS analysis in discovery and identification of serum proteomic patterns of ovarian cancer. BMC Cancer.

[106] Huang H.l., Stasyk T., Morandell S., Dieplinger H., Falkensammer G., Griesmacher A., Mogg M., Schreiber M., Feuerstein I., Huck C. (2006). Biomarker discovery in breast cancer serum using 2-D differential gel electrophoresis/MALDI-TOF/TOF and data validation by routine clinical assays. Electrophoresis.

[107] Zhou Y., Romson J., Emmer Å. (2019). An antibody-free sample pretreatment method for osteopontin combined with MALDI-TOF MS/MS analysis. PLoS ONE.

[108] Oros D., Ceprnja M., Zucko J., Cindric M., Hozic A., Skrlin J., Barisic K., Melvan E., Uroic K., Kos B. (2020). Identification of pathogens from native urine samples by MALDI-TOF/TOF tandem mass spectrometry. Clin. Proteom..

[109] Banach P., Dereziński P., Matuszewska E., Matysiak J., Bochyński H., Kokot Z.J., Nowak-Markwitz E. (2019). MALDI-TOF-MS Analysis in the Identification of Urine Proteomic Patterns of Gestational Trophoblastic Disease. Metabolites.

[110] Hayakawa E., Landuyt B., Baggerman G., Cuyvers R., Lavigne R., Luyten W., Schoofs L. (2013). Peptidomic analysis of human reflex tear fluid. Peptides.

[111] Hardenborg E., Taube A., Hanrieder J., Andersson M., Alm A., Bergquist J. (2009). Protein content in aqueous humor from patients with pseudoexfoliation (PEX) investigated by capillary LC MALDI-TOF/TOF MS. PROTEOMICS-Clin. Appl..

[112] Chaiyarit P., Taweechaisupapong S., Jaresitthikunchai J., Phaonakrop N., Roytrakul S. (2014). Comparative evaluation of 5–15-kDa salivary proteins from patients with different oral diseases by MALDI-TOF/TOF mass spectrometry. Clin. Oral Investig..

[113] Ellias M., Ariffin S., Karsani S., Abdul Rahman M., Senafi S., Megat Abdul Wahab R. (2012). Proteomic Analysis of Saliva Identifies Potential Biomarkers for Orthodontic Tooth Movement. Sci. World J..

[114] Lamy E., Graça G., Costa G., Franco C., Capela e Silva F., Baptista E., Coelho A. (2010). Changes in mouse whole saliva soluble proteome induced by tannin-enriched diet. Proteome Sci..

[115] Fu Y.R., Yi Z.J., Guan S.Z., Zhang S.Y., Li M. (2012). Proteomic analysis of sputum in patients with active pulmonary tuberculosis. Clin. Microbiol. Infect..

[116] Preianò M., Maggisano G., Murfuni M.S., Villella C., Colica C., Fregola A., Pelaia C., Lombardo N., Pelaia G., Savino R. (2018). Rapid Detection and Identification of Antimicrobial Peptide Fingerprints of Nasal Fluid by Mesoporous Silica Particles and MALDI-TOF/TOF Mass Spectrometry: From the Analytical Approach to the Diagnostic Applicability in Precision Medicine. Int. J. Mol. Sci..

[117] Poth A., Deeth H., Alewood P., Holland J. (2008). Analysis of the human casein phosphoproteome by 2-D electrophoresis and MALDI-TOF/TOF MS reveals new phosphoforms. J. Proteome Res..

[118] Van Raemdonck G.A.A., Tjalma W.A.A., Coen E.P., Depuydt C.E., Van Ostade X.W.M. (2014). Identification of protein biomarkers for cervical cancer using human cervicovaginal fluid. PLoS ONE.

[119] Yang H., Zhou B., Prinz M., Siegel D. (2012). Proteomic analysis of menstrual blood. Mol. Cell. Proteom. MCP.

[120] Casado-Vela J., Rodriguez-Suarez E., Iloro I., Ametzazurra A., Alkorta N., García-Velasco J.A., Matorras R., Prieto B., González S., Nagore D. (2009). Comprehensive Proteomic Analysis of Human Endometrial Fluid Aspirate. J. Proteome Res..

[121] Shen X., Liu X., Zhu P., Zhang Y., Wang J., Wang Y., Wang W., Liu J., Li N., Liu F. (2017). Proteomic analysis of human follicular fluid associated with successful in vitro fertilization. Reprod. Biol. Endocrinol. RBE.

[122] Zuberovic A., Wetterhall M., Hanrieder J., Bergquist J. (2009). CE MALDI-TOF/TOF MS for multiplexed quantification of proteins in human ventricular cerebrospinal fluid. Electrophoresis.

[123] Muratovic A., Hanrieder J., Hellman U., Bergquist J., Wetterhall M. (2008). Proteome Profiling of Human Cerebrospinal Fluid: Exploring the Potential of Capillary Electrophoresis with Surface Modified Capillaries for Analysis of Complex Biological Samples. Eur. J. Mass Spectrom..

[124] Mateos Martín J., Lourido L., Fernández-Puente P., Calamia V., Fernández-López C., Oreiro N., Ruiz-Romero C., Blanco F. (2012). Differential protein profiling of synovial fluid from rheumatoid arthritis and osteoarthritis patients using LC-MALDI TOF/TOF. J. Proteom..

[125] Wang F., Chen F.-F., Gao W.-B., Wang H.-Y., Zhao N.-W., Xu M., Gao D.-Y., Yu W., Yan X.-L., Zhao J.-N. (2016). Identification of citrullinated peptides in the synovial fluid of patients with rheumatoid arthritis using LC-MALDI-TOF/TOF. Clin. Rheumatol..

[126] Oliveira M., Oliveira R., Lima A., Andrade E., Abreu J., Oliveira F. (2017). Physical evaluation, morphological and identification of seminal proteins in Santa Ines sheep. Rev. Bras. De Saúde E Produção Anim..

[127] Dietrich M.A., Irnazarow I., Ciereszko A. (2017). Proteomic identification of seminal plasma proteins related to the freezability of carp semen. J. Proteom..

[128] Tian M., Cui Y.-Z., Song G.-H., Zong M.-J., Zhou X.-Y., Chen Y., Han J.-X. (2008). Proteomic analysis identifies MMP-9, DJ-1 and A1BG as overexpressed proteins in pancreatic juice from pancreatic ductal adenocarcinoma patients. BMC Cancer.

[129] Fétaud V., Frossard J.-L., Farina A., Pastor C.M., Bühler L., Dumonceau J.-M., Hadengue A., Hochstrasser D.F., Lescuyer P. (2008). Proteomic profiling in an animal model of acute pancreatitis. Proteomics.

[130] Chang W.C., Huang M.S., Yang C.J., Wang W.Y., Lai T.C., Hsiao M., Chen C.H. (2010). Dermcidin identification from exhaled air for lung cancer diagnosis. Eur. Respir. J..

[131] Matysiak J., Hajduk J., Mayer F., Hebeler R., Kokot Z. (2016). Hyphenated LC-MALDI-ToF/ToF and LC-ESI-QToF approach in proteomic characterization of honeybee venom. J. Pharm. Biomed. Anal..

[132] Chapeaurouge A., Silva A., Carvalho P., McCleary R.J.R., Modahl C.M., Perales J., Kini R.M., Mackessy S.P. (2018). Proteomic Deep Mining the Venom of the Red-Headed Krait, Bungarus flaviceps. Toxins.

[133] Hoffmann H.J., Tabaksblat L.M., Enghild J.J., Dahl R. (2008). Human skin keratins are the major proteins in exhaled breath condensate. Eur. Respir. J..

[134] Hsieh W.Y., Chen M.W., Ho H.T., You T.M., Lu Y.-T. (2007). Identification of differentially expressed proteins in human malignant pleural effusions. Eur. Respir. J. Off. J. Eur. Soc. Clin. Respir. Physiol..

[135] Jabbour R.E., Snyder A.P., Schaudies R.P. (2014). 14-Mass spectrometry-based proteomics techniques for biological identification. Biological Identification.

[136] Kolmeder C., Lähteenmäki K., Wacklin P., Kotovuori A., Ritamo I., Mättö J., Vos W.M.d., Valmu L. (2017). Tandem Mass Spectrometry in Resolving Complex Gut Microbiota Functions.

[137] Zhang R., Zhang Y., Zhang T., Xu M., Wang H., Zhang S., Zhang T., Zhou W., Shi G. (2022). Establishing a MALDI-TOF-TOF-MS method for rapid identification of three common Gram-positive bacteria (Bacillus cereus, Listeria monocytogenes, and Micrococcus luteus) associated with foodborne diseases. Food Sci. Technol..

[138] Lingpeng Z., Xi H., Xue J., Liu H., Xiong C., Nie Z. (2020). MALDI-TOF/TOF Tandem Mass Spectrometry Imaging Reveals Non-uniform Distribution of Disaccharide Isomers in Plant Tissues.

[139] Feucherolles M., Cauchie H.-M., Penny C. (2019). MALDI-TOF Mass Spectrometry and Specific Biomarkers: Potential New Key for Swift Identification of Antimicrobial Resistance in Foodborne Pathogens. Microorganisms.

[140] Fagerquist C.K., Dodd C.E. (2021). Top-down proteomic identification of plasmid and host proteins produced by pathogenic Escherichia coli using MALDI-TOF-TOF tandem mass spectrometry. PLoS ONE.

[141] Clark C.M., Costa M.S., Sanchez L.M., Murphy B.T. (2018). Coupling MALDI-TOF mass spectrometry protein and specialized metabolite analyses to rapidly discriminate bacterial function. Proc. Natl. Acad. Sci. USA.

[142] Lebeau A., Bruyere D., Roncarati P., Peixoto P., Hervouet E., Cobraiville G., Taminiau B., Masson M., Gallego C., Mazzucchelli G. (2022). HPV infection alters vaginal microbiome through down-regulating host mucosal innate peptides used by Lactobacilli as amino acid sources. Nat. Commun..

[143] Conrotto P., Hellman U. (2005). Sulfonation chemistry as a powerful tool for MALDI TOF/TOF de novo sequencing and post-translational modification analysis. J. Biomol. Tech. JBT.

[144] Pekov S., Indeykina M., Popov I., Kononikhin A., Bocharov K., Kozin S., Makarov A., Nikolaev E. (2017). Application of MALDI-TOF/TOF-MS for relative quantitation of α- and β-Asp7 isoforms of amyloid-β peptide. Eur. J. Mass Spectrom..

[145] Koehbach J., Gruber C.W., Becker C., Kreil D.P., Jilek A. (2016). MALDI TOF/TOF-Based Approach for the Identification of d- Amino Acids in Biologically Active Peptides and Proteins. J. Proteome Res..

[146] Franc V., Rehulka P., Medda R., Padiglia A., Floris G., Sebela M. (2013). Analysis of the glycosylation pattern of plant copper amine oxidases by MALDI-TOF/TOF MS coupled to a manual chromatographic separation of glycans and glycopeptides. Electrophoresis.

[147] Irungu J., Go E., Zhang Y., Dalpathado D., Liao H.-X., Haynes B., Desaire H. (2008). Comparison of HPLC/ESI-FTICR MS versus MALDI-TOF/TOF MS for glycopeptide analysis of a highly glycosylated HIV envelope glycoprotein. J. Am. Soc. Mass Spectr..

[148] Scholten A., Visser N., Heuvel R., Heck A. (2006). Analysis of protein-protein interaction surfaces using a combination of efficient lysine acetylation and nanoLC-MALDI-MS/MS applied to the E9:Im9 bacteriotoxin—immunity protein complex. J. Am. Soc. Mass Spectr..

[149] Jagannadham M., Kameshwari D., Pratapa G., Nagaraj R. (2017). Detection of peptides with intact phosphate groups using MALDI TOF/TOF and comparison with the ESI-MS/MS. Eur. J. Mass Spectrom..

[150] Xu C.-F., Lu Y., Ma J., Mohammadi M., Neubert T.A. (2005). Identification of Phosphopeptides by MALDI Q-TOF MS in Positive and Negative Ion Modes after Methyl Esterification*S. Mol. Cell. Proteom..

[151] Söderberg C., Lambert W., Kjellström S., Wiegandt A., Wulff R., Månsson C., Rutsdottir G., Emanuelsson C. (2012). Detection of crosslinks within and between proteins by LC-MALDI-TOFTOF and the software FINDX to reduce the MSMS-data to acquire for validation. PLoS ONE.

[152] Mascini N.E., Teunissen J., Noorlag R., Willems S.M., Heeren R.M.A. (2018). Tumor classification with MALDI-MSI data of tissue microarrays: A case study. Methods.

[153] Everest-Dass A.V., Briggs M.T., Kaur G., Oehler M.K., Hoffmann P., Packer N.H. (2016). N-glycan MALDI Imaging Mass Spectrometry on Formalin-Fixed Paraffin-Embedded Tissue Enables the Delineation of Ovarian Cancer Tissues*. Mol. Cell. Proteom..

[154] Balluff B., Frese C.K., Maier S., Schone C., Kuster B., Schmitt M., Aubele M., Hofler H., Deelder A.M., Heck A. (2014). De novo discovery of phenotypic intratumour heterogeneity using imaging mass spectrometry. J. Pathol..

[155] Tan H., Lim T., Chung M., Lin Q. (2011). iTRAQ™ Labeling Coupled with LC-MALDI Mass Spectrometry for Monitoring Temporal Response of Colorectal Cancer Cells to Butyrate Treatment. Methods Mol. Biol..

[156] Hodgkinson V.C., Agarwal V., Elfadl D., Fox J.N., McManus P.L., Mahapatra T.K., Kneeshaw P.J., Drew P.J., Lind M.J., Cawkwell L. (2012). Pilot and feasibility study: Comparative proteomic analysis by 2-DE MALDI TOF/TOF MS reveals 14-3-3 proteins as putative biomarkers of response to neoadjuvant chemotherapy in ER-positive breast cancer. J. Proteom..

[157] Kang S., Maeng H., Kim B.G., Qing G.M., Choi Y.P., Kim H.Y., Kim P.S., Kim Y., Kim Y.H., Choi Y.D. (2012). In situ Identification and Localization of IGHA2 in the Breast Tumor Microenvironment by Mass Spectrometry. J. Proteome Res..

[158] Chi L.-M., Lee C.-W., Chang K.-P., Hao S.-P., Lee H.-M., Liang Y., Hsueh C., Yu C.-J., Lee I.N., Chang Y.-J. (2009). Enhanced Interferon Signaling Pathway in Oral Cancer Revealed by Quantitative Proteome Analysis of Microdissected Specimens Using 16O/18O Labeling and Integrated Two-dimensional LC-ESI-MALDI Tandem MS*. Mol. Cell. Proteom..

[159] Gawin M., Kurczyk A., Stobiecka E., Frątczak K., Polańska J., Pietrowska M., Widłak P. (2019). Molecular Heterogeneity of Papillary Thyroid Cancer: Comparison of Primary Tumors and Synchronous Metastases in Regional Lymph Nodes by Mass Spectrometry Imaging. Endocr. Pathol..

[160] Panderi I., Perez K., Cao L., Noble L., Lombardo K., Walsh T., Pantazatos D. (2017). Assessment of molecular differentiation in FFPE colon adenocarcinoma tissues using PCA analysis of MALDI IMS spectral data. J. Appl. Bioanal..

[161] Jiang J., Parker C., Hoadley K., Perou C., Boysen G., Borchers C. (2007). Development of an immuno tandem mass spectrometry (iMALDI) assay for EGFR diagnosis. Proteomics. Clin. Appl..

[162] Akpinar G., Kasap M., Canturk N.Z., Zulfigarova M., Islek E.E., Guler S.A., Simsek T., Canturk Z. (2017). Proteomics Analysis of Tissue Samples Reveals Changes in Mitochondrial Protein Levels in Parathyroid Hyperplasia over Adenoma. Cancer Genom. Proteom..

[163] Mocanu C., Iavorschi M., Drochioiu G. (2020). Aluminium Binding to Modified Amyloid-β Peptides: Implications for Alzheimer’s Disease. Molecules.

[164] Khorjestan S., Abtahi B., Siadat S., Motevalli S.m., Rezadoost H., Ghezellou P., Ghassempour A. (2015). Analysis of annulated sea snake venom, Hydrophis Cyanocinctus, using liquid chromatography and MALDI-TOF/TOF. Curr. Proteom..

[165] Rubakhin S.S., Sweedler J.V. (2008). Quantitative Measurements of Cell–Cell Signaling Peptides with Single-Cell MALDI MS. Anal. Chem..

[166] Nachman R., Russell W., Predel R. (2005). MALDI-TOF/TOF Mass Spectrometric Assignment of Leu/Ile in PVK-CAP2b Neuropeptides From Single Neurohemal Organ Preparations of Four Flies.

[167] Jia C., Hui L., Cao W., Lietz C., Jiang X., Chen R., Catherman A., Thomas P., Ge Y., Kelleher N. (2012). High-definition De Novo Sequencing of Crustacean Hyperglycemic Hormone (CHH)-family Neuropeptides. Mol. Cell. Proteom. MCP.

[168] Duffy C., Sorolla A., Wang E., Golden E., Woodward E., Davern K., Ho D., Johnstone E., Pfleger K., Redfern A. (2020). Honeybee venom and melittin suppress growth factor receptor activation in HER2-enriched and triple-negative breast cancer. npj Precis. Oncol..

[169] Gu H., Han S.M., Park K.-K. (2020). Therapeutic Effects of Apamin as a Bee Venom Component for Non-Neoplastic Disease. Toxins.

[170] Nguyen H., Heger Z., Kominkova M., Michálek P., Gumulec J., Guráň R., Pridal A., Fernández C., Hynek D., Adam V. (2014). The Electrochemical and Statistical Evaluation of Isolation of Mellitin and Apamin from Honey Bee (Apis Mellifera) Venom. Int. J. Electrochem. Sci..

[171] Galán J.A., Sánchez E.E., Bashir S., Pérez J.C. (2005). Characterization and identification of disintegrins in Crotalus horridus venom by liquid chromatography and tandem matrix-assisted laser desorption ionization-quadrupole ion trap time-of-flight (MALDI-QIT-TOF) mass spectrometry. Can. J. Chem..

[172] Arruda Macêdo J.K., Fox J.W., de Souza Castro M. (2015). Disintegrins from snake venoms and their applications in cancer research and therapy. Curr. Protein Pept. Sci..

[173] Chen C., Laviolette S.R., Whitehead S.N., Renaud J.B., Yeung K.K.C. (2021). Imaging of Neurotransmitters and Small Molecules in Brain Tissues Using Laser Desorption/Ionization Mass Spectrometry Assisted with Zinc Oxide Nanoparticles. J. Am. Soc. Mass Spectr..

[174] Nayak T., Mandal S.M., Neog K., Ghosh A. (2018). Characterization of a Gloverin-Like Antimicrobial Peptide Isolated from Muga Silkworm, Antheraea assamensis. Int. J. Pept. Res. Ther..

[175] Kumar J., Sharma V.K., Singh D.K., Mishra A., Gond S.K., Verma S.K., Kumar A., Kharwar R.N. (2016). Epigenetic Activation of Antibacterial Property of an Endophytic Streptomyces coelicolor Strain AZRA 37 and Identification of the Induced Protein Using MALDI TOF MS/MS. PLoS ONE.

[176] Chu S., Baker M., Leong G., Letcher R., Gee S., Hammock B., Li Q. (2017). Exploring adduct formation between human serum albumin and eleven organophosphate ester flame retardants and plasticizers using MALDI-TOF/TOF and LC-Q/TOF. Chemosphere.

[177] Ruttkay-Nedecky B., Nejdl L., Gumulec J., Zitka O., Masarik M., Eckschlager T., Stiborova M., Adam V., Kizek R. (2013). The role of metallothionein in oxidative stress. Int. J. Mol. Sci..

[178] Elvis O., Smith J., Clark C., Schlager J., Shih M. (2001). MALDI-ToF/MS as a diagnostic tool for the confirmation of sulfur mustard exposure. J. Appl. Toxicol. JAT.

[179] Alam S., Kumar B., Kamboj D. (2012). Multiplex Detection of Protein Toxins Using MALDI-TOF-TOF Tandem Mass Spectrometry: Application in Unambiguous Toxin Detection from Bioaerosol. Anal. Chem..

[180] Calvano C., Bianco M., Losito I., Cataldi T. (2021). Proteomic Analysisof Food Allergens by MALDI TOF/TOF Mass Spectrometry.

[181] Mohammadi M., Falak R., Mokhtarian K., Khorramizadeh M.R., Kardar G. (2016). Identification and Characterization of Main Allergic Proteins in Cooked Wolf Herring Fish. Iran. J. Allergy Asthma Immunol..

[182] Aiello D., Materazzi S., Risoluti R., Thangavel H., Di Donna L., Mazzotti F., Casadonte F., Siciliano C., Sindona G., Napoli A. (2015). A major allergen in rainbow trout (Oncorhynchus mykiss): Complete sequences of parvalbumin by MALDI tandem mass spectrometry. Mol. BioSystems.

[183] Ballardini N., Nopp A., Hamsten C., Vetander M., Melen E., Nilsson C., Ollert M., Flohr C., Kuehn A., Hage M. (2017). Anaphylactic Reactions to Novel Foods: Case Report of a Child With Severe Crocodile Meat Allergy. Pediatrics.

[184] Piras C., Hale O.J., Reynolds C.K., Jones A.K., Taylor N., Morris M., Cramer R. (2021). Speciation and milk adulteration analysis by rapid ambient liquid MALDI mass spectrometry profiling using machine learning. Sci. Rep..

[185] Wahab N., Abdullah N., Aminudin N. (2014). Characterisation of Potential Antidiabetic-Related Proteins from Pleurotus pulmonarius (Fr.) Quél. (Grey Oyster Mushroom) by MALDI-TOF/TOF Mass Spectrometry. BioMed Res. Int..

[186] Sethy N., Bhardwaj A., Singh V., Sharma R., Deswal R., Bhargava K., Misra K. (2017). Characterization of ganoderma lucidum: Phytochemical and proteomic approach. J. Proteins Proteom..

[187] Matuszewska E., Matysiak J., Rosiński G., Kędzia E., Ząbek W., Zawadziński J., Matysiak J. (2021). Mining the Royal Jelly Proteins: Combinatorial Hexapeptide Ligand Library Significantly Improves the MS-Based Proteomic Identification in Complex Biological Samples. Molecules.

[188] Wang M., Wang Y., Wang J., Lin L., Hong H., Wang D. (2011). Proteome profiles in medaka (*Oryzias melastigma*) liver and brain experimentally exposed to acute inorganic mercury. Aquat. Toxicol..

[189] Tripković T., Charvy C., Alves S., Lolić A.Đ., Baošić R.M., Nikolić-Mandić S.D., Tabet J.C. (2013). Identification of protein binders in artworks by MALDI-TOF/TOF tandem mass spectrometry. Talanta.

[190] Cleland T., Schroeter E. (2018). A Comparison of Common Mass Spectrometry Approaches for Paleoproteomics. J. Proteome Res..

